# *Aspergillus ustus* Infections among Transplant Recipients

**DOI:** 10.3201/eid1203.050670

**Published:** 2006-03

**Authors:** Anil A. Panackal, Alexander Imhof, Edward W. Hanley, Kieren A. Marr

**Affiliations:** * Fred Hutchinson Cancer Research Center; Seattle, Washington, USA;; † University of Washington Medical Center, Seattle, Washington, USA;; ‡ University Hospital, Zürich, Zürich, Switzerland

**Keywords:** Aspergillus ustus, antifungal drug resistance, emerging pathogen, outbreak, transplantation, research

## Abstract

*Aspergillus ustus* is a mold that rarely infects humans; only 15 systemic cases have been reported. We report the first outbreak of invasive infection caused by *A. ustus* among hematopoietic stem cell transplant (HSCT) recipients. Six patients with infections were identified; 3 infections each occurred in both 2001 and 2003. Molecular typing by using randomly amplified polymorphic DNA (RAPD) and antifungal drug susceptibility testing were performed on clinical and environmental isolates recovered from our hospital from 1999 to 2003. The highest overall attack rate in HSCT patients was 1.6%. The overall death rate was 50%, and death occurred within 8 days after diagnostic culture collection. Clinical isolates exhibited decreased susceptibility to antifungal drugs, especially azoles. RAPD and phylogenetic analysis showed genetic similarity between isolates from different patients. Based on the clustering of cases in space and time and molecular data, common-source acquisition of this unusual drug-resistant species is possible.

## Abstract 2

This is the first report of clustered *Aspergillus ustus* causing systemic disease in transplant patients.

Invasive aspergillosis (IA) has become a devastating opportunistic fungal infection among immunocompromised hosts, with a 357% increase in death rates reported in the United States from 1980 to 1997 (*1*). The most common cause of IA is *Aspergillus fumigatus* (*2*). However, in recent years, IA has been increasingly caused by non-*fumigatus Aspergillus* species. For example, at the Fred Hutchinson Cancer Research Center in Seattle, the proportion of infections caused by non-*fumigatus Aspergillus* species increased during the latter 1990s. Most of these infections were caused by *A. flavus*, *A. nidulans*, *A. terreus*, and *A. niger* (*3*).

*Aspergillus ustus* is a group of filamentous hyalohyphomycetes consisting of 5 species: *A. ustus*, *A. puniceus*, *A. panamensis*, *A. conjunctus*, and *A. deflectus*. Members of this group are rare human pathogens; only 15 cases of systemic infection have been reported in the literature since 1970, and more than half of these occurred in the past 10 years (Table A1) (*4**-**17*). Infections caused by *A. ustus* may be of particular concern, as the organisms exhibit low susceptibility to multiple antifungal drugs, and outcomes have been uniformly poor (Table A1). Recognition of invasive infections that occurred in 2 clusters of hematopoietic stem cell transplant (HSCT) recipients in our institution prompted us to perform a more thorough clinical investigation and environmental sampling to identify potential sources of acquisition.

## Methods

### Case Identification and Environmental Surveillance

Recognition of time-clustered cases in 2003 prompted us to do this retrospective study and epidemiologic investigation. Cases of infection caused by *A. ustus* were identified by review of microbiology and infection control records available from 1993 to 2003. Charts were reviewed for clinical data (demographics, underlying disease, transplantation characteristics, antifungal therapies, radiographic and laboratory studies, and outcome). Cases were classified as proven, probable, or possible according to consensus criteria published by the European Organization for Research and Treatment of Cancer/Invasive Fungal Infections Cooperative Group and the National Institute of Allergy and Infectious Diseases Mycoses Study Group (*18*). The Fred Hutchinson Cancer Research Center institutional review board approved this study.

The hospital is a large tertiary care facility that houses patients with HSCT on the top 2 floors (the northeast wings of the seventh and eighth floors). A spot map depicting case-patient location and timeline relating location to time of diagnosis was created. Information on timing of construction activities and airflow information was obtained from hospital engineering and infection control personnel. An attack rate was estimated among the potentially exposed HSCT patients using as the denominator the number of patients who were admitted for HSCT from July through October 2001 and March through September 2003, the at-risk periods when cases occurred.

### Environmental Sampling

Based on the spot map, environmental air sampling of patient hospital rooms was performed, and environmental isolates were obtained. An air particle sampler (SAS Super 100, PBI International, Milan, Italy) was used to collect ambient "dust" to the 0.3-μm size. Samples (0.5 m^3^) were cultured on inhibitory mold agar plates (Remel IMA plates, Lenexa, KS, USA). Organisms were identified to the species level by using standard morphologic criteria for *A. ustus*. Isolates were stored at -70°C.

### Molecular Typing

Molecular typing of *A. ustus* clinical and environmental isolates was performed by randomly amplified polymorphic DNA (RAPD) analysis by using *A. ustus* ATCC 1041, NRRL 275, and *Candida parapsilosis* for outgroup comparison (*19*). DNA templates were purified from ≈50 mg cells, resuspended in phosphate-buffered saline (PBS), treated with Lyticase 10 μg/mL (Sigma Chemical Co., St. Louis, MO, USA) for 1 h at 37°C, and then digested with Proteinase K 10 μg/mL (Sigma Chemical Co.). Mixtures were subjected to 3 cycles of freeze-thaw in liquid nitrogen, alternating with vortexing with 0.2 g glass beads. Genomic DNA was isolated with the DNeasy Tissue Kit (Qiagen, Hilden, Germany) according to the manufacturer's instructions. The RAPD reactions were run under conditions optimized for each primer (Table 1) by using a PerkinElmer 9700 thermal cycler (PerkinElmer, Cetus, CT, USA). PCR products underwent electrophoresis in 1.8% agarose gels, were stained with ethidium bromide, and images were obtained by using an Alpha Imager (Alpha Innotech Corporation, San Leandro, CA, USA). Only bands that possessed one-tenth the integrated intensity of the 1,650-bp band of the molecular marker (4 ng) (area under the curve [AUC] = 1,132) were defined as positive bands for subsequent band relational analysis. The band patterns from each gel with each primer were analyzed by using tools for population genetics analysis (TFPGA) (unpub. data). Cluster analysis was performed by the unweighted pair group mean with arithmetic average (UPGMA) method (*20*). Bootstrapping was performed with 1,000 tree comparisons with averages by using TFPGA. Band patterns of >95% similarity were classified as identical.

**Table 1:** Conditions for *Aspergillus ustus* DNA amplification.

	Primers				
	Ustus 1	R151	RPO2	OPA10	OPA20
Primer sequence	5´-GTA TTG CCC T-3´	5´-GCT GTA GTG T-3´	5´-GCG ATC CCC A-3´	5´-GTG ATC GCA G-3´	5´-GTT GCG ATC C-3´
Primer concentration	0.8 pmol/L	1.0 pmol/L	1.0 pmol/L	0.4 pmol/L	1.0 pmol/L
MgCl_2_	1.8 mmol/L	2.2 mmol/L	3.0 mmol/L	1.8 mmol/L	2.0 mmol/L
Template concentration	0.025 ng/50μL	0.5 ng/50μL	0.012 ng/50μL	0.03 ng/50μL	0.1 ng/50μL
Annealing temperature	32°C	32°C	34°C	32°C	32°C
Annealing time	1.5 min	1.5 min	1.5 min	1.5 min	1.5 min

### Antifungal Drug Susceptibility Testing

Antifungal drug susceptibility testing of *A. ustus* isolates was performed by using a microbroth dilution assay, as described by the National Committee for Clinical Laboratory Standards or the filamentous fungi (M38-A) for itraconazole (Janssen, Titusville, NJ, USA), voriconazole (Pfizer, New York, NY, USA), and amphotericin B (Bristol-Myers Squibb Co., Princeton, NJ, USA) (*21*). Susceptibility (minimal effective concentration) to caspofungin (Merck Research Laboratories, Rahway, NJ, USA) was determined by using a microbroth dilution assay in antibiotic 3 (AM3) media, as described previously (*22*).

## Results

### Outbreak Cases

We identified 2 clusters of A. ustus infection among HSCT recipients in our hospital during the study. The first occurred from July to October 2001 (3 probable lung infections: patients 1, 2, and 3). The second occurred from March to September 2003 (1 proven skin infection [likely disseminated from lung] and 2 probable lung infections: patients 4, 5, and 6) (Table A1); 1 lung transplant recipient was colonized with *A. ustus* while in the hospital (data not shown).

The median age of patients was 59 (range 29 -63) years; 5 (83.3%) were male; median neutropenia duration 15 (range 4-22) days; 5 (83.3%) patients had graft-versus-host disease that required therapy; 5 (83.3%) patients had received mold-active antifungal drugs prophylactically (itraconazole, n = 4) or for a prior diagnosis (voriconazole, n = 1). The median time of diagnosis after transplantation was 222 (range 60-1,295) days (n = 5). Three (50%) of the 6 patients died, all within 8 days of diagnostic culture collection.

### Epidemiologic Investigation

Estimating that 382 patients were admitted for HSCT during the at-risk period, the highest overall attack rate was 1.6%, which is above the baseline rate of infection with *A. ustus* at our institution (0%). No changes in laboratory processing or mold identification methods occurred during the study. Of note, construction of a new surgery pavilion occurred outside our hospital building beginning in July 2001 and ending in December 2003. Airflow to hospital rooms in which the patients resided passed through multiple filters (blanket filter, pre-filter, 95% filter, HEPA filter).

**Figure 1:**
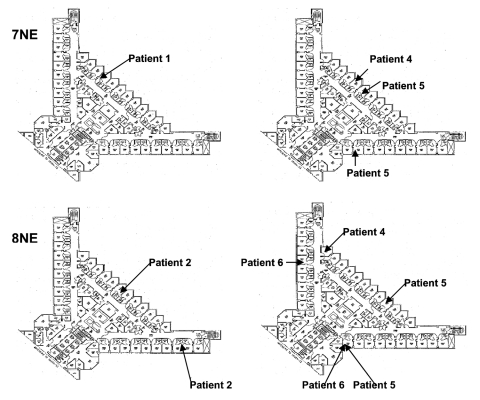
Spot map illustrating case-patient location on the northwestern wing of the eighth floor (8NE) and the seventh floor (7NE) from the 2001 (left panel) and 2003 outbreaks (right panel) at the time of case diagnosis. Patient 3 was in the outpatient clinic at the time of diagnosis and is, therefore, not marked on this inpatient spot map. Patient 5 and 6 resided in the same room at different times. Patients 2, 4, 5, and 6 were moved to a variety of rooms around the time of diagnosis as indicated by their location in multiple rooms.

The spot map and time line showed that cases clustered mainly along 2 corridors on 2 floors, 1 directly above the other, around the time of diagnosis. In the 2001 and 2003 outbreaks, all case-patients resided in the same or adjacent rooms before diagnosis (Figure 1).

Environmental air sampling performed 2 months after the last case occurred in 2003 found no *A. ustus* isolates in the rooms of HSCT patients. One environmental *A. ustus* isolate was obtained from the carpeted floor of the hall near the room in which the colonized lung transplant recipient resided. The same bronchoscope was used to evaluate each patient; however, it was cleaned after each examination. Also, several patients who were not found to have *A. ustus* on bronchoalveolar lavage underwent bronchoscopic examination before case-patients, suggesting that cross-contamination was unlikely.

### Analyses of Isolates

**Figure 2:**
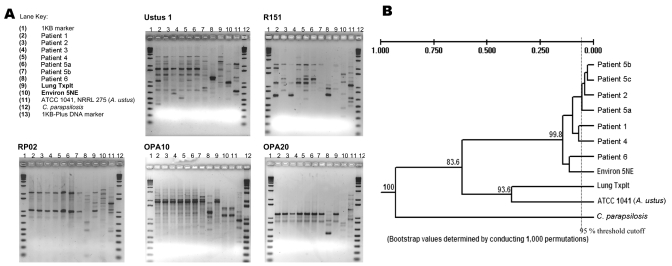
Molecular typing of *Aspergillus ustus* isolates by using random amplification of polymorphic DNA. The isolate from patient 3 was not viable on subculturing and, as such, was not available for molecular analysis. Gel images (A) and composite dendrogram (B) are shown.

Eleven *A. ustus* isolates were available for analysis. One patient (patient 3) did not have a viable isolate stored, and 1 patient (patient 5) had 3 isolates recovered during the course of infection. A total of 73 bands were resolved from the 11 *A. ustus* isolates (Figure 2). The isolates recovered from the 5 HSCT were genetically similar. Three isolates from patient 5 were genetically most similar to the isolate from patient 2. At the time of his diagnosis and death, patient 2 resided in a room directly adjacent to and above the room of patient 5, albeit 2 years earlier (Figure 1). Similarly, the isolate from patient 1 was genetically most similar to that of patient 4; patients 1 and 4 resided in adjacent rooms, also separated by a period of 2 years. Of note, the lung transplant patient appeared to be colonized with a strain of *A. ustus* that was genetically as distant from the patient isolates as the wild-type ATCC strain. Antifungal drug susceptibility testing of clinical isolates demonstrated relatively high MICs to all antifungal drugs tested (Table 2).

**Table 2:** Antifungal drug susceptibility testing of *Aspergillus ustus* isolates

Isolate	Amphotericin B*	Voriconazole*	Itraconazole*	Caspofungin†
Patient 1	2.0	8.0	4.0	2.0
Patient 2	2.0	4.0	2.0	2.0
Patient 4	2.0	8.0	4.0	4.0
Patient 5a	1.0	4.0	2.0	8.0
Patient 5b	1.0	8.0	8.0	4.0
Patient 5c	2.0	8.0	4.0	2.0
Patient 6	2.0	8.0	8.0	2.0
Lung transplant	2.0	4.0	1.0	2.0
Environment	4.0	8.0	4.0	2.0

*MIC _100_, μg/mL. †Minimum effective concentration, μg/mL.

## Discussion

We report the first outbreak of disease caused by an unusual fungal pathogen, *A. ustus*, a mold that has rarely caused invasive disease in humans. This observation is important, given the possibility of common-source acquisition of a potentially antifungal drug–resistant organism.

All members of the *A. ustus* complex have similar shape with subtle differences. Macroscopically, the colonies appear drab olive to dull brown or gray and woolly with occasional dark purple or yellow exudates. Microscopically, the conidia are large (3.0–4.5 μm) and are rough-walled. Elongate and irregular-shaped Hülle cells that are resistant to desiccation may also be produced (*23*). *A. ustus* is toxigenic and produced several mycotoxins such as austdiol, austin, austocystin A, and sterigmatocystin (*24**–**27*). Although these toxins may be medically important, the quantities of toxin produced in the environment may not be significant (*28**,**29*). The spectrum of disease reported due to *A. ustus* includes onychomycosis, otitis media, primary cutaneous infection, endocarditis, pneumonia, and disseminated infection, the latter cases occurring largely among immunocompromised hosts such as HSCT recipients. All previously reported cases occurred sporadically in diverse medical centers (Table A1). Many reported cases have been either primary cutaneous disease or disseminated infection, however, we cannot draw firm conclusions regarding the types of infections this organism causes because of the high likelihood of reporting bias. The relative pathogenicity of this *Aspergillus* species has not been well studied.

In the 6 HSCT patients described in this article, infection developed late after HSCT, with a high proportion of deaths (*30**,**31*). These patients also possessed classic risk factors for IA in that most had graft-versus-host disease that required corticosteroid and other immunosuppressive therapy (*30*). Overall death rates of patients with *A. ustus* infection was high in this cohort, as in previous cases (*4**–**15*). Whether death was attributable to the fungal infection, coinfections, or underlying diseases is unclear.

A common source for the *A. ustus* infections appears possible, since case-patients clustered in space and time, and a high degree of genetic similarity was noted between isolates from case-patients. Since these patients resided in rooms within close proximity, common source acquisition (e.g., air, water, or surface) is credible. Common source acquisition may not be precluded by case isolate separation in time as *Aspergillus* conidia are resistant to harsh conditions, surviving in the environment for many years in dormant phase (*32*). However, the environmental niche of this fungus is not known. Patients were in and out of the hospital after transplantation, so infection could have been acquired in the environment. We also cannot rule out the possibility that other clinical factors (e.g., changes in hosts or antifungal drug administration) selected for specific *A. ustus* isolates in the patients.

The results of molecular analyses suggest genetic similarity of isolates recovered from patients. Although discriminatory power of RAPD analyses has some limitations (*33*), the composite analysis demonstrated large separations between patient isolates and the control ATCC strain. Our study is limited by the lack of local environmental *A. ustus* isolates available for genetic comparison. Although the clinical isolates appear different from the ATCC strain, the genetic similarity of case strains may represent a strain common to our local environment. Also, these analyses are limited by our lack of knowledge concerning *A. ustus*'s modes of reproduction. Specifically, genomic rearrangement with recombination, which has been postulated to occur in several species of *Aspergillus*, may increase the variation observed between related strains (*34*).

Our investigation was limited by constraints in conducting retrospective analyses. More timely environmental sampling may have captured more environmental *A. ustus* isolates (*32**,**35*). For example, swabbing of dust-ridden surfaces may have indicated the underlying air quality in terms of fungal spores in the preceding months when infection may have occurred. In the absence of substantial air disturbances, *A. ustus* spores would be more likely to quickly settle in such areas, given their large size and relatively decreased buoyancy. Construction, a well-known environmental risk factor for IA (*36*), was ongoing outside the hospital during the time of these outbreaks. We cannot comment on the role of water as a source of infection, which has been reported in multiple hospitals (*32**,**35*). Determining the source of infection is further complicated in that a combination of inoculum effect and underlying host immunosuppression make calculating the incubation period problematic. Thus, the source of *A. ustus* infection among our patients, and whether the infections were nosocomial or community acquired, remains unknown.

These outbreaks of *A. ustus* infections may be of infection control importance, as the clinical isolates exhibited low susceptibilities to multiple antifungal drugs, as was reported previously (*12**,**17*). Although we do not know breakpoints of *A. fumigatus* resistance, results of prior studies suggest that infection with organisms requiring high MICs of amphotericin or itraconazole is associated with poor clinical outcomes (*37**,**38*). Most of our patients received mold-active azole drugs before diagnosis as either prophylaxis or therapy for a previous infection with *A. fumigatus*. Similarly, *A. ustus* was recently reported to cause "breakthrough" infection during administration of voriconazole and caspofungin (*17*). Drug exposure may select for colonization or infection with resistant isolates or facilitate acquired resistance within a colonizing strain. The latter may occur in *A. fumigatus* isolates exposed to azole antifungal agents (*39**,**40*). In this cohort, several patients who received the combination regimen of voriconazole and caspofungin had *A. ustus* infection resolve; whether this resolution was due to drug synergy in treating relatively resistant organisms is worthy of further consideration.

*A. ustus* is rare; however, it may be emerging as a cause of systemic disease among immunocompromised hosts in the appropriate setting. A combination of factors, including severity of underlying host immunosuppression and common source acquisition, likely played a role in the reported outbreaks. Active laboratory, environmental, and clinical-based surveillance for *A. ustus* has been implemented at our hospital based on the results of this investigation; no additional isolates have been identified subsequently. Such intensive monitoring may show similar outbreaks in other facilities. This study also emphasizes the importance of establishing microbial diagnoses to the species level; information obtained is important for infection control and, possibly, to guide antifungal therapies. More studies will be necessary to determine the clinical consequence of antifungal resistance in *A. ustus* isolates.

## Appendix

**Table A1:** *Aspergillus ustus* infections reported during the last 10 years*

Pt./ref.	Age (y)	Sex	Underlying condition	Diagnosis/site	Coinfection	Clinical indications	Immuno-suppression	Management	Outcome
(*4*)	41	M	Aortic valve replacement	Femoral artery thrombus, endocarditis	None	Endocarditis with emboli	N/A	Ampho B and flucytosine, surgery	Cure, survived
(*5*)	57	M	Skin burns	Skin†	Bacterial pneumonia	Sharp, demarcated white areas over dorsal hands and arms	Prednisone emulsion	Ampho B cream	Failure, died
(*6*)	50	M	Rheumatic heart disease, prosthetic valve replacement	Femoral artery thrombus; endocarditis	None	Endocarditis with emboli	N/A	Ampho B and flucytosine, surgery	Cure, died
(*7*)	72	M	Cardiovascular disease	Lung‡	Bacterial pneumonia	Multilobar pneumonia, thyroid, lung, kidney, peritoneum abscesses	N/A	None	Failure, died§
(*8*)	62	M	Cirrhosis, orthotopic liver transplant	Skin*‡	None	Multiple crusted nodules, plaques	Cyclosporin and tacrolimus for rejection	Ampho B and terbinafine cream	Cure, died
(*9*)	9	M	Allogeneic HSCT	Lung	None	Fever, chest pain, nodular pulmonary opacity	N/A	Ampho B	Failure, died§
(*10*)	64	F	Chronic obstructive pulmonary disease	Skin*	None	Erythematous plaque with pustules, erosions	Prednisone	Itraconazole	Failure, died§
(*11*)	46	F	Allogeneic HSCT	Lung, disseminated to skin, heart, thyroid‡	CMV, parainfluenza pneumonia; BK virus hemorrhagic cystitis	Bilateral pulmonary consolidation, skin lesion	Myeloablative conditioning	Itraconazole → liposomal ampho B	Failure, died§
(*12*)	38	M	Allogeneic HSCT	Lung‡	None	Seizures, bronchopneumonia	Myeloablative conditioning, GVHD	Ampho B spray → ampho B	Failure, died
(*13*)	77	F	Frontotemporal astrocytoma	Skin*	None	Ulcerative erythematous plaque, suppuration	Dexamethasone	Itraconazole + KMnO_4_ soaks	Failure, died
(*14*)	19	F	Allogeneic HSCT	Skin*	Bacteremia	Skin ulcers	Myeloablative conditioning, GVHD	Ampho B	Failure, died§
(*15*)	43	M	Leukemia	Lung	None	Neutropenic fever, pulmonary infiltrates	cytarabine and idarubicin	Ampho B → voriconazole surgery	Cure, died
(*16*)	N/A	N/A	Allogeneic HSCT	Lung, skin	N/A	N/A	Chronic GVHD	Liposomal ampho B	N/A
(*17*)	29	M	Allogeneic HSCT	Lung, skin, brain dissemination	None	Pulmonary infiltrates and skin lesions	Myeloablative conditioning, GVHD	Ampho B → liposomal ampho B and caspofungin	Cure, died
(*17*)	17	M	Allogeneic HSCT	Lung disseminated to eye, brain, skin	None	Retinitis and skin lesions	GVHD	Voriconazole and ampho B	Failure, died
This article (case 1)	42	M	Allogeneic HSCT	Proven lung	Bacterial pneumonia	Pleural-based nodules	GVHD	Liposomal ampho B and caspofungin	Failure, Died §
This article (case 2)	29	M	Allogeneic HSCT	Probable lung	*A. fumigatus* pneumonia	Cough, dyspnea, and pulmonary nodules	GVHD	Liposomal ampho B and caspofungin	Failure, died
This article (case 3)	62	M	Leukemia	Probable lung	None	Pulmonary consolidation	Fludarabine, Rituximab, Corticosteroids	Liposomal ampho B → voriconazole and caspofungin	Cure, survived
This article (case 4)	59	M	Allogeneic HSCT	Probable lung	Influenza B	Fever, chills, and dyspnea, pulmonary consolidation	GVHD	Voriconazole and caspofungin	Cure, survived
This article (case 5)	48	F	Allogeneic HSCT	Proven lung, skin dissemination	CMV pneumonitis	Pulmonary infiltrates and erythematous papular skin lesions	GVHD	Voriconazole and caspofungin → liposomal ampho B	Failure, died§
This article (case 6)	69	M	Allogeneic HSCT	Probable lung	CMV antigenemia	Dyspnea and pulmonary opacity	GHVD	Voriconazole and Caspofungin	Cure, survived

*GVHD, graft-versus-host disease; N/A, data not applicable or not reported; HSCT, hematopoietic stem cell transplant; CMV, cytomegalovirus; Ampho B, amphotericin V; KMnO_4_, potassium permanganate. †Primary cutaneous infection. ‡Nosocomial acquired infection. §Mortality attributed to *A. ustus* infection.
